# Cost of Illness of Multiple Sclerosis - A Systematic Review

**DOI:** 10.1371/journal.pone.0159129

**Published:** 2016-07-13

**Authors:** Olivia Ernstsson, Hanna Gyllensten, Kristina Alexanderson, Petter Tinghög, Emilie Friberg, Anders Norlund

**Affiliations:** 1 Department of Learning, Informatics, Management and Ethics, Karolinska Institutet, SE-171 77 Stockholm, Sweden; 2 Department of Clinical Neuroscience, Karolinska Institutet, SE-171 77 Stockholm, Sweden; 3 The Swedish Red Cross University College, Stockholm, Sweden; University of Utah, UNITED STATES

## Abstract

**Background:**

Cost-of-illness (COI) studies of Multiple Sclerosis (MS) are vital components for describing the economic burden of MS, and are frequently used in model studies of interventions of MS. We conducted a systematic review of studies estimating the COI of MS, to compare costs between studies and examine cost drivers, emphasizing generalizability and methodological choices.

**Material and method:**

A literature search on studies published in English on COI of MS was performed in PubMed for the period January 1969 to January 2014, resulting in 1,326 publications. A mapping of studies using a bottom-up approach or top-down approach, respectively, was conducted for the 48 studies assessed as relevant. In a second analysis, the cost estimates were compared between the 29 studies that used a societal perspective on costs, human capital approach for indirect costs, presenting number of patients included, time-period studied, and year of price level used.

**Results:**

The mapping showed that bottom-up studies and prevalence approaches were most common. The cost ratios between different severity levels within studies were relatively stable, to the ratio of 1 to 2 to 3 for disability level categories. Drugs were the main cost drivers for MS-patients with low disease severity, representing 29% to 82% of all costs in this patient group, while the main cost components for groups with more advanced MS symptoms were production losses due to MS and informal care, together representing 17% to 67% of costs in those groups.

**Conclusion:**

The bottom-up method and prevalence approach dominated in studies of COI of MS. Our findings show that there are difficulties in comparing absolute costs across studies, nevertheless, the relative costs expressed as cost ratios, comparing different severity levels, showed higher resemblance. Costs of drugs were main cost drivers for less severe MS and informal care and production losses for the most severe MS.

## Introduction

Multiple sclerosis (MS) is a degenerative neurological disease of chronic nature [1, 2], often with unpredictable course [3]. MS cause both healthcare use and reduction of work capacity [4, 5]. For estimates of the economic burden of a disease, cost-of-illness (COI) studies are often implemented [6] which is also the case for MS [7]. There are several literature reviews of COI of MS, however, they either were published before year 2006 [8–11], focused on specific geographical areas [12, 13], were limited to intangible costs due to MS [14], or focused on specific treatment or drugs [15, 16]. In addition, a series of studies by Karampampa and colleagues [17] are now available that were not published before the two most recent literature reviews [7, 18]. There is, thus, a need for an updated systematic review of COI of MS. Important methodological aspects of COI studies that are essential to consider in systematic reviews include: the perspective of the analysis (e.g., societal), the scope of costs measured (e.g., direct, indirect, and intangible costs), the use of an incidence-based approach (including patients from time of disease onset or disease diagnosis) or prevalence approach (including patients at all stages of the disease) [19], as well as any other targeting of patients (such as including only those with relapsing-remitting MS). Another important consideration is whether a COI study uses a top down (TD) or a bottom up (BU) approach [4]. The latter concerns if estimates of costs are based on patient reports (BU) or on other types of information, e.g., from administrative registers of costs (TD).

Direct costs include inpatient care, outpatient care, drugs, diagnostics, surgical interventions, nursing care, social services, and patients´ travel costs in order to get to health care. Indirect costs are losses of production due to short- or long-term sickness absence, disability pension (in some countries called early retirement on medical grounds or incapacity benefit), early old-age pension due to health problems, permanent losses due to premature death, and sometimes time spent by next of kin to care for the patient. Intangible costs concern humanitarian losses due to, for instance, pain, anxiety, and suffering. It has been reported that the economic burden of MS includes medical and non-medical direct costs, indirect costs from increased morbidity, early mortality, and impact on family and friends, and intangible costs [20].

TD calculations usually rely on population-based data for a specific diagnosis and associated resource use and are often restricted to hospital admissions, reductions in productive work, and other resource use that can be identified in registers. BU calculations are commonly based on enquiries to individuals having the disease, and may thus include questions on e.g., informal care and transportation not often found in registers. The results of a BU study can start from a subpopulation and be extrapolated to the total population.

This variation in methods used in COI studies makes comparison of results between studies difficult, and concerns have been raised of the generalizability of results, also in the MS context [9, 21]. Thus, the aim of this systematic review was to compare COI estimates for MS between studies, overall and by level of severity of MS, and to examine cost drivers for the estimates, emphasizing studies with results that were generalizable to all patients with MS in the population of e.g., a country. To do this, patterns in methods used for estimating the COI of MS needed to be explored to enhance comparisons between studies.

## Methods

**Fig 1:**
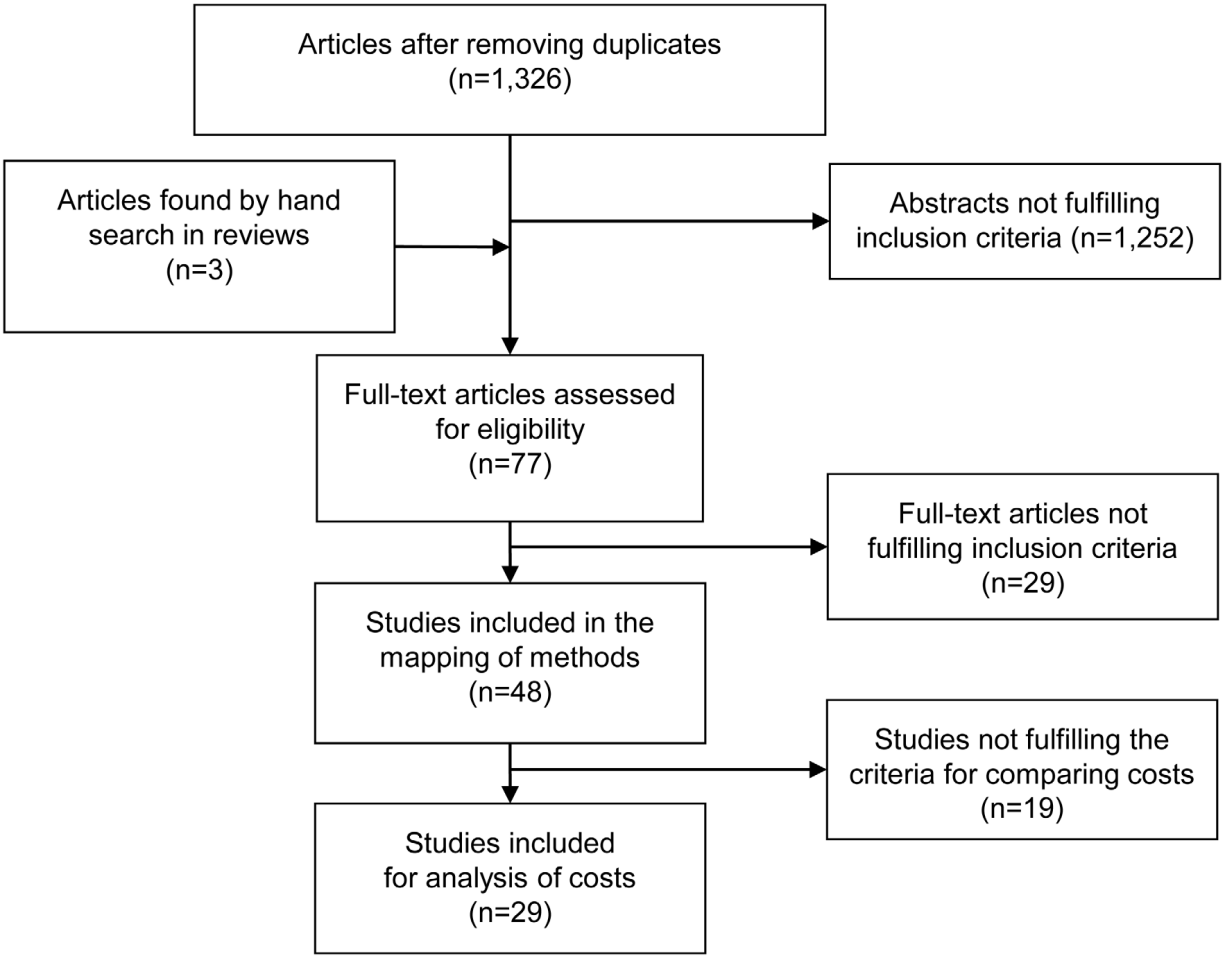
Flow chart.

We conducted a systematic review following the PRISMA statement [22], including the following steps. Published studies on COI of MS were searched in PubMed with a supplementary search in the Health Economic Evaluations Database (HEED) for additional studies. The search terms used were Multiple Sclerosis AND (costs OR cost of illness OR economic costs OR economic burden OR economic impact OR economic status OR economic deprivation OR economic pressure OR burden of disease OR social consequences), limited to studies published in English between January 1969 to January 24^th^ 2014. The search strategy using the term “Multiple sclerosis” in combination with other search terms in separate searches resulted in a total of 2,621 studies, of which 1,326 studies remained after excluding duplicates (Fig 1). Full-text versions of articles were reviewed independently by OE and AN, and were retrieved if at least one of the reviewers considered the study to be relevant. Reference lists of eligible articles were hand searched for additional studies. The flow chart covering the search of literature is described in Fig 1.

Criteria were used for inclusion of studies, where any disagreement between reviewers was solved by discussions among the authors to reach consensus. The inclusion criteria were: research published in English in a peer reviewed journal, containing information on prevalence- or incidence-based cost data for MS, from OECD-countries. Intangible costs were not considered in this review. This resulted in 74 abstracts identified as possible relevant studies. Three additional publications were found through hand search in the identified reviews, resulting in a total of 77 studies that were examined in full text.

To identify relevant publications for inclusion in the mapping of COI studies on MS, the 77 identified publications were assessed in full-text for relevance. Twenty-nine of the studies did not meet the inclusion criteria, and reasons for exclusion were: three studies based on compensation data, twelve reviews, seven extrapolations, two summaries of previous studies, and one drug intervention study, as well as three studies without cost data and one in another language than English. Thus, 48 publications met our inclusion criteria and these were mapped by two authors [OE, AN] according to how methodological approaches were implemented: cost components included, TD or BU approach, incidence or prevalence approach.

Among the included studies, an additional assessment was performed by OE and AN to identify a sub-sample of publications with similar methodology that enabled comparisons of study results. Twenty-nine publications, with similar methodology and providing the information needed, was identified, i.e., studies having a societal perspective approach, including both direct and indirect costs, using human capital approach for indirect costs, and providing comprehensive data on number of patients, time-period studied, currency, and year of price level. Reason for not being included in this sub-sample is provided in S1 Table. Data on study characteristics and included cost categories were extracted from the 29 studies. In order to compare study results, costs per patient (overall and by severity of MS) were transformed using Purchasing Power Parities (PPP) for Gross domestic product to USD [23]. The cost data of studies using year of price level before 2011 were inflated by 1 percent annually in order to calculate a common end value for the year 2011. In two of the included studies [24, 25], results were presented in an alternate currency (Euro for non-Euro countries) and recalculations based on the exchange rates given in the articles were made in order to follow the principle of using PPP for each country. In two other studies [26, 27], exchange rates were not reported, why the PPP rate for the EU area was chosen although this concerned non-euro countries. For studies not presenting annual costs, transformations were made to 12 months basis, assuming that there were no seasonal variations in resource use.

The level of MS disability can be evaluated and presented according to the Expanded Disability Status Scale (EDSS), ranging from 0 (normal) to 10 (death due to MS) [28]. Thus, for further comparison, data on costs for patients with different levels of severity were extracted from twelve publications reporting costs by EDSS. The costs for different levels of EDSS were transformed and inflated to 2011 values, and were furthermore compared as costs ratios where the categorization of EDSS I of each study was the comparator of EDSS II and III for the same study.

## Results

After exclusion of duplicates, 1,326 studies remained, and applications of criteria resulted in 77 studies examined in full-text. A total of 29 studies were excluded before the final version of the mapping, i.e., 48 included studies. [1–5, 24–27, 29–67], of which 42 studies were categorized as mainly using a BU approach. The traditional TD approach based on register data was used in the remaining six studies (Table 1). Five studies were categorized as examining a special aspect, i.e., incidence for one year [62], intangible costs [63], the cost of relapse [64, 67], and cost for MS-patients with spasticity [60].

**Table 1:** Mapping of relevant studies (n = 48) regarding having a bottom-up or top-down approach and by type of costs included in the analyses.

	Mainly bottom up	Mainly top down
**Direct costs**	Berto [2], 2011	Gilden [65], 2011
	Bourdette [29], 1993	
	Patti [30], 2011	
	Carton [31], 1998	
**Indirect costs**	Coleman [32] 2013	
**Direct + indirect costs**	Amato [3], 2002	Asche [1], 1997
	Auty [33], 1998*	Henriksson [4], 1998
	Berg [25], 2006*	Jennum [24], 2012*
	Casado [34], 2006*	Blumhardt [66], 1996
	Dusankova [26], 2012*	
	Grima [5], 2000	
	Henriksson [35], 2001*	
	Holmes [36], 1995	
	Johansson [37], 2012*	
	Karampampa [38–42], 2012*	
	Karampampa [43], 2013*	
	Kobelt [44–52], 2006*	
	Kobelt [53], 2009*	
	McCrone [54], 2008*	
	Murphy [55], 1998	
	Oleen-Burkey [56], 2012	
	Orlewska [57], 2005*	
	Palmer [58], 2013*	
	Reese [59], 2011*	
	Svendsen [27], 2012*	
	Svensson [60], 2013	
	Taylor [61], 2007*	
**Special aspect**	Asche [62], 2010	Parisé [67], 2013
	Casado [63], 2007	
	Zettl [64], 2013	

* Included in the further analysis displayed in Table 2

Twenty-nine studies of those included in the mapping were further compared regarding cost of MS [24–27, 33–35, 37–54, 57–59, 61]. All these 29 studies used a prevalence approach and all studies but one [24] mainly used a BU approach (Table 2). There were 17 countries covered by the included studies. Ten of the studies were conducted by the same Swedish group of authors [25, 44–53] and seven studies were done by another group with connections to Sweden [37–43], resulting in that 17 (59%) of the studies were conducted by two Swedish research centers.

**Table 2:** Summary of each of the 29 studies included in the analysis of comparing costs.

Reference	Study population		Cost-of-illness methodology			Cost estimates		
First author, Year, Country	Source population	Definition of MS	Method of calculation	Data sources	Specification of costs	Direct costs	Indirect cost	Total costs
**Auty** [33] 1998, Canada	MS patients recruited from MS centers (n = 198; response rate not given)	MS diagnosis according to Poser criteria	Bottom up, Prevalence	Patients and family via Case Report Form; Clinical charts and summaries on medical history; Price lists; Market prices; Statistics on wages	Direct medical costs, direct non-medical costs, indirect costs	Mean annual cost per patient: Direct medical Mild $2,250; Moderate $1,969; Severe $7,233	Mean annual cost per patient:	Mean annual cost per patient $29,109
	EDSS ≤2.5 (n = 62); EDSS 3–6 (n = 68); EDSS ≥6.5 (n = 68)					Direct non-medical Mild $912; Moderate $1,663; Severe $7,787	Mild $11,360; Moderate $18,068; Severe $22,002	Mild $14,522; Moderate $21,698; Severe $37,024
**Berg** [25] 2006, Sweden	MS patients recruited from patients´ organization register (n = 1,339, 64% response rate)	MS according to patients’ own estimates	Bottom up, Prevalence	Patients via questionnaire; Price lists; Personal communication; Statistics on wages	Direct health care costs, direct non-medical costs, indirect costs, intangible costs	Mean annual cost per patient: Direct medical €15,186	Mean annual cost per patient €17,151	Mean annual cost per patient €53,601
	EDSS 0–3 (29%); EDSS 4–6.5 (45.5%); EDSS 7–9 (25.2%)	RRMS Progressive MS				Direct non-medical €21,264		
**Casado** [34] 2006, Spain	MS patients recruited from one MS center (n = 200, 44% response rate)	n/a	Bottom up, Prevalence	Patients via questionnaire; Inpatient records; Price lists from health care; Statistics on wages; Market prices for pharmaceuticals	Direct costs, indirect costs, informal care costs	Mean annual cost per patient €15,860	Mean annual cost per patient €8,412	Mean annual cost per patient €24,272
	EDSS 0 (n = 23); EDSS 1–3 (n = 107); EDSS 3.5–5.5 (n = 42); EDSS 6–7 (n = 17); EDSS 7.5–9.5 (n = 11)	RRMS, SPMS, PPMS				Stage 1 €8,706; Stage 2 €12,221; Stage 3 €18,724; Stage 4 €24,037; Stage 5 €37,062	Stage 1 €5,621; Stage 2 €6,616; Stage 3 €9,596; Stage 4 €17,161; Stage 5 €15,779	Stage 1 €14,327; Stage 2 €18,837; Stage 3 €27,869; Stage 4 €41,198; Stage 5 €52,841
**Blahova Dusankova** [26] 2012, Czech Republic	MS patients recruited from MS centers (n = 1,027, 89% response rate)	Diagnosis of MS according to the 2005 revised McDonald criteria	Bottom up, Prevalence	Patients via questionnaire; Medical records; Price lists from health care	Direct health care costs, direct non-medical costs, indirect costs	Mean annual cost per patient: Direct medical €6,296	Mean annual cost per patient €5,519	Mean annual cost per patient €12,272
	EDSS 0–3.5 (n = 579); EDSS 4–6.5 (n = 246); EDSS 7–9.5 (n = 87)	RRMS, SPMS, PPMS/RPMS				Direct non-medical €457		
**Henriksson** [35] 2001, Sweden	MS patients recruited from one MS center (n = 413, 76% response rate)	Definite clinical MS according to Poser criteria	Bottom up, Prevalence	Patients via questionnaire; Medical records; Price lists from health care; Community price lists; Personal communication; Statistics on wages	Direct costs, indirect costs, intangible costs	Annual cost per patient €35,728	Annual cost per patient €17,518	Annual cost per patient €53,246
	EDSS ≤3 (n = 126); EDSS 3.5–6 (n = 121); EDSS ≥6.5 (n = 162)	RRMS, SPMS, PPMS						
**Johansson** [37] 2012, France	MS patients recruited from MS centers (n = 248, 61% response rate)	MS diagnosis (ICD-10; G35, ICD-9; 340)	Bottom up, Prevalence	Patients via questionnaire; Price lists from health care and insurance payer; Published literature; Statistics on wages	Direct medical costs, direct non-medical costs, indirect costs	Mean annual cost per patient: Direct medical €15,445; Mild €13,242; Moderate €19,845; Severe €19,491	Mean annual cost per patient €3,022	Mean annual cost per patient €20,738
	EDSS 0–3 (n = 164); EDSS 4–6.5 (n = 69); EDSS 7–9 (n = 11)	RRMS, SPMS, PPMS				Direct non-medical €2,271; Mild €1,051; Moderate €3,967; Severe €16,049	Mild €1,715; Moderate €5,440; Severe €8,448	Mild €16,009; Moderate €29,252; Severe €43,988
**Karampampa** [38] 2012, Canada	MS patients recruited from MS centers (n = 241, response rate not given)	MS diagnosis (ICD-10; G35, ICD-9; 340)	Bottom up, Prevalence	Patients via questionnaire; Price lists from public sources; Price lists from private providers; Statistics on wages	Direct medical costs, direct non-medical costs, indirect costs	Mean annual cost per patient: Direct medical Mild $19,837; Moderate $14,058; Severe $9,478	Mean annual cost per patient	Mean annual cost per patient $37,672
	EDSS 0–3 (n = 146); EDSS 4–6.5 (n = 89); EDSS 7–9 (n = 5)	RRMS, SPMS, PPMS				Direct non-medical Mild $3,848; Moderate $12,712; Severe $44,022	Mild $7,151; Moderate $19,853; Severe $24,480	Mild $30,836; Moderate $46,622; Severe $77,981
**Karampampa** [39] 2012, Spain	MS patients recruited from MS centers (n = 324, 99% response rate)	MS diagnosis (ICD-10; G35, ICD-9; 340)	Bottom up, Prevalence	Patients via questionnaire; Published literature; Price lists from public sources; Statistics on wages	Direct medical costs, direct non-medical costs, indirect costs	Mean annual cost per patient: Direct medical Mild €14,594; Moderate €18,924; Severe €15,845	Mean annual cost per patient	Mean annual cost per patient €29,401
	EDSS 0–3 (n = 209); EDSS 4–6.5 (n = 105); EDSS 7–9 (n = 10)	RRMS, SPMS, PPMS				Direct non-medical Mild €1,386; Moderate €12,441; Severe €22,910	Mild €4,680; Moderate €12,583; Severe €20,592	Mild €20,659; Moderate €43,948; Severe €59,347
**Karampampa** [40] 2012, Germany	MS patients recruited from MS centers (n = 244, 63% response rate)	MS diagnosis (ICD-10; G35, ICD-9; 340)	Bottom up, Prevalence	Patients via questionnaire; Price lists from public sources; Published literature; Statistics on wages	Direct medical costs, direct non-medical costs, indirect costs	Mean annual cost per patient: Direct medical Mild €16,954; Moderate; €17,841; Severe €30,348	Mean annual cost per patient	Mean annual cost per patient
	EDSS 0–3 (n = 164); EDSS 4–6.5 (n = 69); EDSS 7–9 (n = 11)	RRMS, SPMS, PPMS				Direct non-medical Mild €1,163; Moderate €12,373; Severe €22,926	Mild €3,057; Moderate €9,710; Severe €10,996	Mild €21,174; Moderate €39,923; Severe €64,270
**Karampampa** [41] 2012, Italy	MS patients recruited from MS centers (n = 251, 83% response rate)	MS diagnosis (ICD-10; G35, ICD-9; 340)	Bottom up, Prevalence	Patients via questionnaire; Price lists from public sources; Regional tariffs; Published literature; Statistics on wages	Direct medical costs, direct non-medical costs, indirect costs	Mean annual cost per patient: Direct medical Mild €21,418; Moderate €30,507; Severe €13,646	Mean annual cost per patient	Mean annual cost per patient €26,041
	EDSS 0–3 (n = 203); EDSS 4–6.5 (n = 44); EDSS 7–9 (n = 4)	RRMS, SPMS, PPMS				Direct non-medical Mild €447; Moderate €5,634; Severe €15,826	Mild €596; Moderate €5,185; Severe €10,120	Mild €22,461; Moderate €41,327; Severe €39,592
**Karampampa** [42] 2012, UK	MS patients recruited from MS centers (n = 194, 33% response rate)	MS diagnosis (ICD-10; G35, ICD-9; 340)	Bottom up, Prevalence	Patients via questionnaire; Price lists from public sources; Published literature; Statistics on wages	Direct medical costs, direct non-medical costs, indirect costs	Mean annual cost per patient: Direct medical Mild £6,714; Moderate £8,101; Severe £6,059	Mean annual cost per patient	Mean annual cost per patient £21,512
	EDSS 0–3 (n = 77); EDSS 4–6.5 (n = 110); EDSS 7–9 (n = 7)	RRMS, SPMS, PPMS				Direct non-medical Mild £1,913; Moderate £10,299; Severe £41,242	Mild £3,214; Moderate £7,494; Severe £11,717	Mild £11,841; Moderate £25,894; Severe £59,018
**Karampampa** [43] 2013, The Netherlands	MS patients recruited from MS centers (n = 263, response rate not given)	MS diagnosis (ICD-10; G35, ICD-9; 340)	Bottom up, Prevalence	Patients via questionnaire; Price lists; Published literature; Statistics on wages	Direct medical costs, direct non-medical costs, indirect costs	Mean annual cost per patient: Direct medical €12,265; Mild €11,274; Moderate €13,668; Severe €13,978	Mean annual cost per patient €20,284	Mean annual cost per patient €47,173
	EDSS 0–3 (n = 122); EDSS 4–6.5 (n = 112); EDSS 7–9 (n = 29)	RRMS, SPMS, PPMS				Direct non-medical €14,624; Mild €4,951; Moderate €14,967; Severe €52,303	Mild €14,714; Moderate €22,421; Severe €34,188	Mild €30,938; Moderate €51,056; Severe €100,469
**Kobelt** [44] 2006, The Netherlands	MS patients recruited from MS centers (n = 1,549, 52% response rate)	MS according to patients’ own estimates	Bottom up, Prevalence	Patients via questionnaire; Price lists from health care; Statistics on wages; WTP/QALY	Direct medical costs, direct non-medical costs, indirect costs including friction costs, intangible costs	Mean annual cost per patient: Direct medical €8,371	Mean annual cost per patient €13,476	Mean annual cost per patient €29,423
	EDSS 0–3 (47.9%); EDSS 4–6.5 (39.6%); EDSS 7–9.5 (11.2%)	RRMS, Progressive MS				Direct non—medical €7,576	*Friction cost €611*	*Friction cost €16*,*600*
**Kobelt** [45] 2006, Switzerland	MS patients recruited from patients´ organization register (n = 1,101, 44% response rate)	MS according to patients’ own estimates	Bottom up, Prevalence	Patients via questionnaire; Price lists from health care; Personal communication; Statistics on wages; WTP/QALY	Direct medical costs, direct non-medical costs, indirect costs, intangible costs	Mean annual cost per patient: Direct medical €11,237	Mean annual cost per patient €15,928	Mean annual cost per patient €41,873
	EDSS 0–3 (38.3%); EDSS 4–6.5 (35.8%); EDSS 7–9 (22.8%); EDSS 8–9 (14.3%)	RRMS, Progressive MS				Direct non-medical €14,708		
**Kobelt** [46] 2006, UK	MS patients recruited from patients´ organization register (n = 2,048, 16% response rate)	MS according to patients’ own estimates	Bottom up, Prevalence	Patients via questionnaire; Price lists from health care; Published literature; Market prices; Statistics on wages; WTP/QALY	Direct medical costs, direct non-medical costs, indirect costs, intangible costs	Mean annual cost per patient: Direct medical £6,810	Mean annual cost per patient £11,174	Mean annual cost per patient £30,263
	EDSS 0–3 (21.3%); EDSS 4–6.5 (59.6%); EDSS 7–9 (19.1%)	RRMS, Progressive MS				Direct non-medical £12,298		
**Kobelt** [47] 2006, Austria	MS patients recruited from patients´ organization register (n = 1,019, 34% response rate)	MS according to patients’ own estimates	Bottom up, Prevalence	Patients via questionnaire; Price lists from health care; Personal communication; Statistics on wages; WTP/QALY	Direct medical costs, direct non-medical costs, indirect costs, intangible costs	Mean annual cost per patient: Direct medical €17,302	Mean annual cost per patient €14,657	Mean annual cost per patient €40,309
	EDSS 0–3 (40.6%); EDSS 4–6.5 (35.6%); EDSS 7–9.5 (22.2%)	RRMS, Progressive MS				Direct non-medical €8,351		
**Kobelt** [48] 2006, Germany	MS patients recruited from MS centers (53%) and from one database (47%) (n = 2,793, 38% response rate)	MS according to patients’ own estimates	Bottom up, Prevalence	Patients via questionnaire; Price lists from health care; Personal communication; Market prices; Statistics on wages; WTP/QALY	Direct medical costs, direct non-medical costs, indirect costs, intangible costs	Mean annual cost per patient: Direct medical €17,165	Mean annual cost per patient €16,911	Mean annual cost per patient €39,998
	EDSS 0–3 (47.4%); EDSS 4–6.5 (35.6%); EDSS 7–9.5 (12%)	RRMS, Progressive MS				Direct non-medical €5,922		
**Kobelt** [49] 2006, Italy	MS patients recruited from patients´ organization register (n = 921, 52% response rate)	MS according to patients’ own estimates	Bottom up, Prevalence	Patients via questionnaire; Price lists from health care; Personal communication; Statistics on wages; WTP/QALY	Direct medical costs, direct non-medical costs, indirect costs, intangible costs	Mean annual cost per patient: Direct medical €11,111	Mean annual cost per patient €11,310	Mean annual cost per patient €38,845
	EDSS 0–3 (31.3%); EDSS 4–6.5 (47.2%); EDSS 7–9.5 (19.6%)	RRMS, Progressive MS				Direct non-medical €16,424		
**Kobelt** [50] 2006, Belgium	MS patients recruited from MS centers (n = 799, 38% response rate)	MS according to patients’ own estimates	Bottom up, Prevalence	Patients via questionnaire; Price lists from health care; Statistics on wages; WTP/QALY	Direct medical costs, direct non-medical costs, indirect costs, intangible costs	Mean annual cost per patient: Direct medical €12,020	Mean annual cost per patient €11,604	Mean annual cost per patient €32,466
	EDSS 0–3.5 (45.5%); EDSS 4–6.5 (32.2%); EDSS 7–9.5 (19.7%)	RRMS; Progressive MS				Direct non-medical €8,842		
**Kobelt** [51] 2006, Spain	MS patients recruited from patients´ organization register (n = 1,848, 32% response rate)	MS according to patients’ own estimates	Bottom up, Prevalence	Patients via questionnaire; Price lists from health care; Statistics on wages; WTP/QALY	Direct medical costs, direct non-medical costs, indirect costs, intangible costs	Mean annual cost per patient: Direct medical €12,142	Mean annual cost per patient €8,775	Mean annual cost per patient €33,456
	EDSS 0–3 (36.1%); EDSS 4–6.5 (44.8%); EDSS 7–9 (17.7%)	RRMS, Progressive MS				Direct non-medical €12,540		
**Kobelt** [52] 2006, US	MS patients randomly selected from one register (n = 1,909, 48% response rate)	MS according to patients’ own estimates	Bottom up, Prevalence	Patients via questionnaire; Price lists from public sources; Personal communication; Statistics on wages; WTP/QALY	Direct medical costs, direct non-medical costs, indirect costs, intangible costs	Mean annual cost per patient $29,634	Mean annual cost per patient $17,581	Mean annual cost per patient $47,215
	EDSS ≤3.5 (34.8%); EDSS 4–6 (42.7%); EDSS ≥6.5 (22.1%)	PPMS, RRMS, SPMS						
**Kobelt** [53] 2009, France	MS patients recruited from patients´ organization register (n = 1,355, 34% response rate)	MS according to patients’ own estimates	Bottom up, Prevalence	Patients via questionnaire; Price lists from health care; Statistics on wages	Direct costs, indirect costs	Mean annual cost per patient €23,654	Mean annual cost per patient €20,730	Mean annual cost per patient €44,384
	EDSS 0–3 (n = 529); EDSS 4–5 (n = 315); EDSS 6–7 (n = 354); EDSS 8–9 (n = 136)	RRMS, Progressive MS						
**McCrone** [54] 2008, UK	MS patients recruited from patients´ organization register (n = 1,942, 49% response rate)	MS according to patients’ own estimates	Bottom up, Prevalence	Patients via questionnaire; Price lists from health care; Published literature; Statistics on wages	Direct costs (service costs), indirect costs (lost employment)	Mean 6.monthly cost £8,397	Mean 6.monthly cost £4,240	Mean 6.monthly cost £12,655
	GNDS 0–9 (n = 192); GNDS 10–19 (n = 694); GNDS 20–29 (n = 734); GNDS 30–39 (n = 265); GNDS ≥40 (n = 43)	RRMS, SPMS, PPMS, Benign MS						
**Orlewska** [57] 2005, Poland	MS patients recruited from MS centers (n = 148, response rate not given)	Definite MS according to Poser criteria	Bottom up, Prevalence	Patients via questionnaire; Price lists from health care; Market prices; Statistics on wages	Direct costs, indirect costs	Mean cost per patient per 5 months	Mean cost per patient per 5 months	Mean cost per patient per 5 months
	EDSS <3.5 (n = 57); EDSS 4–6 (n = 56); EDSS >6.5 (n = 35)	RRMS, SPMS				Mild 4,069 PLN; Moderate 5,399 PLN; Severe 6,010 PLN	Mild 6,886 PLN; Moderate 10,204 PLN; Severe 12,454 PLN	Mild 10,954 PLN; Moderate 15,603 PLN; Severe 18,464 PLN
**Palmer** [58] 2013, Australia	MS patients recruited from register (n = 712, 28% response rate)	Self-reported MS	Bottom up, Prevalence	Patients via questionnaire and diary; Price lists from health care; Statistics on wages	Direct personal costs, direct community/ governmental costs, nursing home and equivalent costs, informal care, indirect costs	Mean annual cost per patient: Personal AUD 3,697; Community AUD 10,721; Nursing home AUD 4,384; Informal care AUD 6,857	Mean annual cost per patient AUD 23,286	Mean annual cost per patient AUD 48,945
	EDSS 1–3; EDSS 4–6; EDSS 6.5–9							
**Reese** [59] 2011, Germany	MS patients recruited from one MS center (n = 144, 77% response rate)	Definite MS according to McDonald diagnostic criteria	Bottom up, Prevalence	Patients via questionnaire; Price lists from health care; Price lists from companies; Published literature; Statistics on wages	Direct medical costs, indirect costs	Mean cost per patient per 3 months €5,483	Mean cost per patient per 3 months €4,846	Mean cost per patient per 3 months €10, 329
	EDSS 0–1.5; EDSS 2–3.5; EDSS 4–5.5; ESSS 6–8.5	RRMS, SPMS, PPMS						
**Svendsen** [27] 2012, Norway	MS patients from one patient organization register and data from national registers (n = 423, 80% response rate)	Definite MS according to Poser criteria	Bottom up/Top down, Prevalence	Patients via questionnaires; Price lists from health care; Medical records; Registers; Statistics on cost of labor	Direct costs, indirect costs	Annual cost to the society €171,387,000	Annual cost to the society €,267,588,000	Mean annual cost per patient €65,037
	EDSS 0–3 (43.5%); EDSS 4–6.5 (43%); EDSS 7–9 (13.5%)	RRMS, PPMS/SPMS						Annual cost to the society €438,975,000
**Taylor** [61] 2007, Australia	MS patients recruited from one MS center (n = 100, response rate not given)	Definite MS according to Poser and Rose criteria, and reclassified according to McDonald criteria	Bottom up, Prevalence	Patients via questionnaire; Data sources for costs not given	Direct costs, indirect costs	Mean annual cost per patient AU$20,396	Mean annual cost per patient AU$15,085	Mean annual cost per patient AU$35,481
	EDSS 0–2.5 (n = 30); EDSS 3–4.5 (n = 29); EDSS 5–6.5 (n = 22); EDSS ≥7 (n = 19)	RRMS, SPMS, PPMS				EDSS 0–2.5 AU$18,568; EDSS 3–4.5 AU$15,504; EDSS 5–6.5 AU$20,159; EDSS ≥ 7 AU$31,025		
**Jennum** [24] 2012, Denmark	MS patients from a national database (n = 10,849)	MS diagnosis (ICD-10)	Top down, Prevalence	Registers on use and costs of health care; Social statistics data	Direct costs, indirect costs, social transfers	Mean annual cost per patient €3,465	Mean annual cost per patient €11,110	Mean annual cost per patient €14,575

The cost categories that were most frequently reported in the 29 included studies are presented in Table 3. All studies included direct costs for inpatient care and costs for drugs, and all studies but one [58] explicitly reported about including direct costs for outpatient care. Costs related to specialists other than those mentioned were, e.g., opticians, speech therapists, psychiatrists, and acupuncturists. The inclusion of other direct costs varied between studies. Among the 29 studies, 25 studies included costs for informal care, 21 reported nursing home costs, and 22 included home help services. Concerning indirect costs, all studies reported short-term work absence, while long-term and permanent reductions in productive work were reported in most, but not all, studies. Permanent reductions in productive work were commonly called early retirement due to MS in the studies (Table 3). Only one study included indirect costs due to premature death [27], while another study stated it was excluded since there was no higher risk of premature death for patients with MS compared to the rest of the population [35]. Costs that were less frequently reported in the studies were e.g., child care, social services and workplace adaptions. As can be seen from Table 3 costs data used in the included studies were based on data with differences of levels of specification. For example, studies by Auty [33] and by Taylor [61] had few specified data, and included only short term absence as regards indirect costs, as compared to the high specifications of data and inclusion also of long term sickness absence in the studies by Kobelt [25, 44–51] and Karampampa [37–43]. The difference of estimated cost per patient corresponded to almost 50%, i.e. 28 575 USD (average for Auty and Taylor) compared to 42 567 USD (average for Kobelt and Karampampa).

**Table 3:** The most frequently reported cost categories in the 29 included studies. The resource use indicated in the table is reported as in the original publication, there is thus a variation in the level of aggregation in information from the different sources.

	Auty [33] 1998	Berg [25] 2006	Casado [34] 2006	B Dusankova [26] 2012	Henriksson [35] 2001	Johansson [37] 2012	Karampampa [38–43] 2012–2013	Kobelt [44–53] 2006	McCrone [54] 2008	Orlewska [57] 2005	Palmer [58] 2013	Reese [59] 2011	Svendsen [27] 2012	Taylor [61] 2007	Jennum [24] 2012
	n = 198	n = 1,339	n = 200	n = 1,027	n = 413	n = 248	n = 1,517	n = 15,342	n = 1,942	n = 148	n = 712	n = 144	n = 423	n = 100	n = 10,849
Inpatient care	X	X	X	X	X	X	X	X	X	X	X	X	X	X	X
Outpatient care	X	X	X	X	X	X	X	X	X	X		X	X	X	X
Rehabilitation		X	X	X	X	X	X	X		X	X	X	X		
General practitioner		X	X	X	X	X	X	X	X			X	X		X
Nurse		X			X	X	X	X	X				X		
Neurologist		X		X	X	X	X	X				X	X		
Physiotherapist		X		X	X	X	X	X	X	X		X	X		
Occupational therapist		X			X	X	X	X				X	X		
Psychologist		X		X	X	X	X	X				X	X		
Other specialists		X		X	X	X	X	X	X	X		X	X		X
Drugs	X	X	X	X	X	X	X	X	X	X	X	X	X	X	X
Tests	X	X	X	X		X	X	X	X	X	X			X	
Home care/ Home visits		X		X	X			X		X	X		X		
Informal care		X	X	X	X	X	X	X	X	X	X		X		
Nursing home		X				X	X	X	X		X		X		
Home help		X		X	X	X	X	X	X					X	
Home or car modifications		X	X	X	X	X	X	X	X	X	X	X	X	X	
Walking aids		X		X	X	X	X	X				X	X	X	
Other investments		X	X	X	X	X	X	X	X	X	X	X	X	X	
Transportations	X	X	X	X				X		X			X	X	
Short-term absence	X ^1^	X	X	X	X	X	X	X	X	X		X	X	X	X ^2^
Long-term absence		X	X	X	X	X	X	X	X	X		X	X		X ^2^
Early retirement		X	X	X	X	X	X	X	X	X ^2^	X ^2^^,^^3^	X	X	X ^2^	X ^2^

^1^ days missed from work during 3 months

^2^ lost employment/unemployment/reduced labor supply

^3^ based on asking participants whether they are employed or not

**Fig 2:**
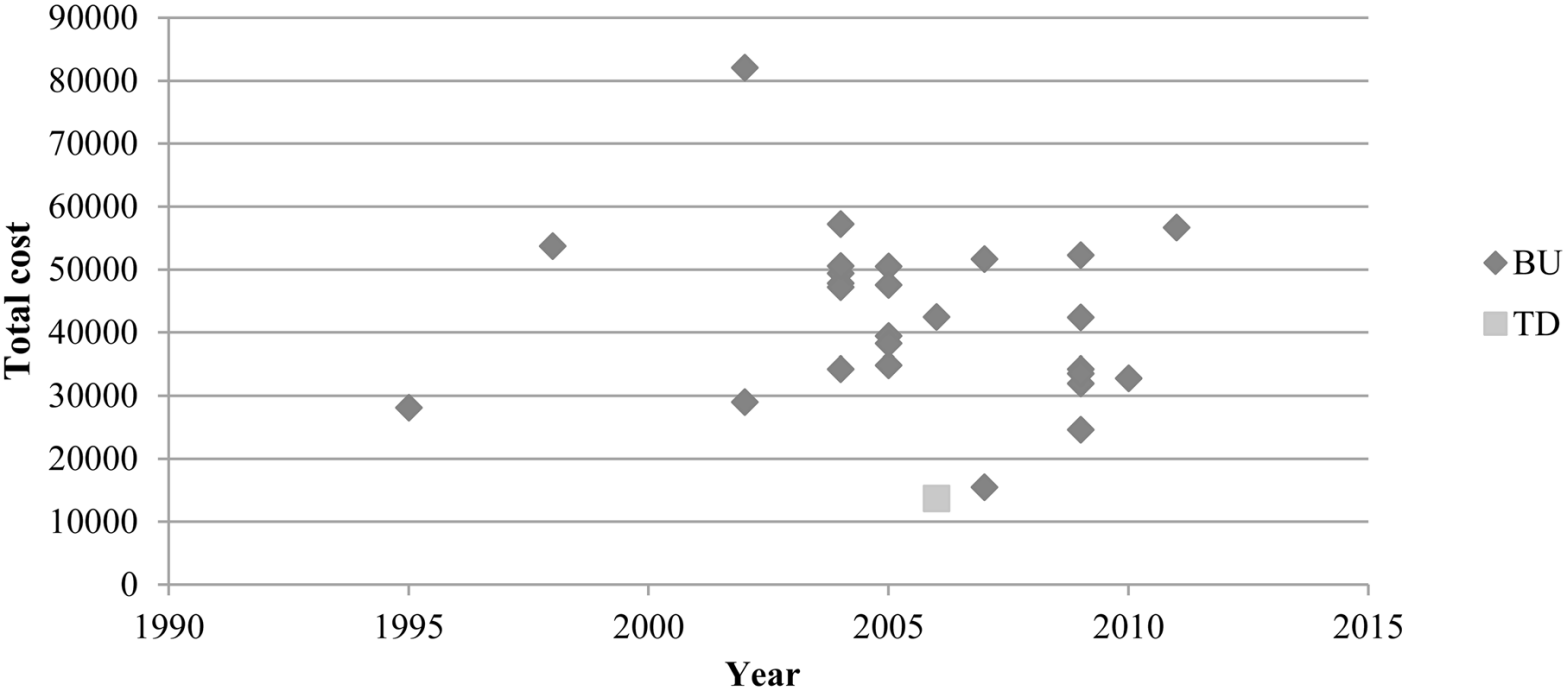
Cost per patient and year, discounted at 1% until 2011.

Per patient costs inflated to end values for the year 2011 are presented in Table 4. After re-calculating the costs per patient, costs were up to six times higher for the studies using the BU approach than that presented in the study using a TD approach (Fig 2). An analysis of linear regression showed a non-significant association (i.e., p<0.05) between year of publication and total cost per patient (r = 0.154, p = 0,44). In comparison of the BU studies, costs per patient differed up to five times between the lowest and highest estimate. Aside from the two studies reporting the highest and lowest cost estimates, costs differed up to two times between the remaining studies.

**Table 4:** Presentation of costs for the year 2011 after transforming costs to US dollars using Purchasing Power Parities (PPP), by inflating 1% annually.

First author, Publication year, Method of calculation	Year of costing	Currency	Total direct + indirect costs	PPP rate	Total costs transformed to USD using PPP, per patient and year	Recalculated with 1% inflation rate, present value 2011
**Top down**						
Jennum [24]^1^ 2012	2006	DKK	108 684	8.31	13 055	13 721
**Bottom up**						
Auty [33] 1998	1995	CDN dollar ($)	29 100	1.21	23 977	28 115
Berg [25]^2^ 2006	2004	SEK	486 354	9.11	53 392	57 243
Casado [34] 2006	2004	Euro (€)	24 272	0.76	31 956	34 261
Dusankova [26] ^3^ 2012	2007	Euro (€)	12 272	0.82	14 897	15 502
Henriksson [35] 2001	1998	SEK	442 476	9.37	47 231	53 753
Karampampa [38] 2012	2009	CAN dollar ($)	37 672	1.20	31 324	31 953
Karampampa [37] 2012	2009	Euro (€)	20 738	0.86	24 180	24 666
Karampampa [41] 2012	2009	Euro (€)	26 041	0.78	33 541	34 215
Karampampa [39] 2012	2009	Euro (€)	29 401	0.71	41 600	42 437
Karampampa [42] 2012	2009	Pound (£)	21 512	0.65	32 922	33 583
Karampampa [43] 2013	2011	Euro (€)	47 173	0.83	56 719	56 719
Kobelt [44] 2006	2004	Euro (€)	29 423	0.91	32 833	34 852
Kobelt [45] 2006	2005	CHF	64 850	1.74	37 211	39 500
Kobelt [46] 2006	2005	Pound (£)	30 263	0.64	47 570	50 497
Kobelt [47] 2006	2004	Euro (€)	40 309	0.87	46 077	49 401
Kobelt [48] 2006	2004	Euro (€)	39 998	0.90	44 592	47 809
Kobelt [49] 2006	2005	Euro (€)	38 845	0.87	44 822	47 580
Kobelt [50] 2006	2004	Euro (€)	32 466	0.90	36 089	38 310
Kobelt [51] 2006	2004	Euro (€)	33 456	0.76	44 047	47 225
Koblt [52] 2006	2004	US dollar ($)	47 215	1.00	47 215	50 621
Kobelt [53] 2009	2007	Euro (€)	44 384	0.89	49 661	51 678
McCrone [54] 2008	2006	Pound (£)	12 655	0.63	40 429	42 491
Palmer [58] 2013	2010	AU dollar ($)	48 945	1.51	32 497	32 822
Reese [59] 2011	2009	Euro (€)	10 329	0.81	51 254	52 284
Svendsen [27]^3^ 2012	2002	Euro (€)	65 037	0.87	75 049	82 080
Taylor [61] 2007	2002	AU$	35 481	1.336	26 548	29 035

^1^ transformed from EUR to DKK with exchange rate (EUR 1 = DKK 7,45) stated in article

^2^ transformed from EUR to SEK with exchange rate (EUR 1 = SEK 9,0736) stated in article

^3^ ‘Euro area’ used in PPP transformations, whereas no exchange rate was stated in article

Twenty-seven out of the 29 included studies presented costs per EDSS level in tables or figures, of which twelve presented costs by three categories of severity levels that we considered to be possible to extract [26, 33, 35, 37–43, 57, 58]. The levels of EDSS I, II, and III represent the categorization of different severities of MS, or case mix of MS, and this was reflected in the costs per patient that increased with higher levels of EDSS (Table 5). Although absolute costs per patient varied highly between the studies, i.e., for EDSS II corresponding to 17,765 USD PPP [26] compared to 61,388 USD PPP [43], the costs ratios per patient (EDSS II and III compared to I) varied much less, i.e., 1.42 vs. 1.65, for Blahova Dusankova et al. [26] and Karampampa et al. [43], respectively (Table 5). The coefficient of variation, defined as standard deviation compared to the mean, was 0.15 and 0.38, for EDSS II and III, respectively, for all 12 studies including EDSS estimates of costs. Concerning the mildest severity categorization, hereafter called EDSS I, seven out of ten studies that specified costs per category identified MS treatment as the main cost driver [26, 37–42]. The cost driver varied more between studies in the moderate severity group, EDSS II, where it was identified as drugs or MS treatment [37, 39–41], permanent reductions in productive work [26, 38, 43], other classifications of indirect costs [57, 58], and informal care [42]. Four out of ten studies [38, 40–42] identified informal care as the main cost driver for individuals in the most severe group, EDSS III, whereas four studies identified production losses due to permanent reductions in productive work [26, 37, 39, 43] and two identified other types of indirect costs [57, 58].

**Table 5:**
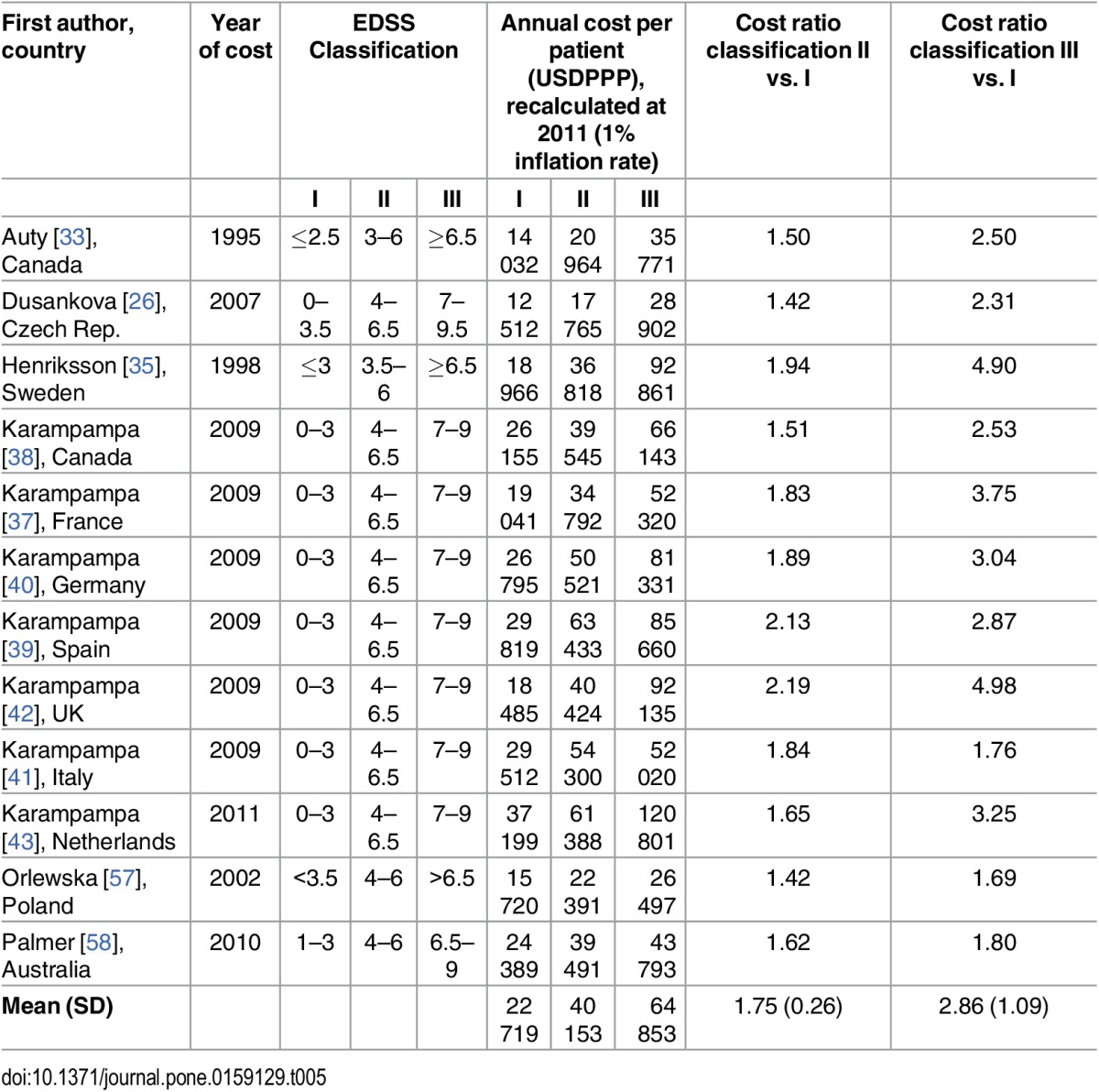
Annual cost per patient by EDSS classification group and cost ratios.

First author, country	Year of cost	EDSS Classification			Annual cost per patient (USDPPP), recalculated at 2011 (1% inflation rate)			Cost ratio classification II vs. I	Cost ratio classification III vs. I
		I	II	III	I	II	III		
Auty [33], Canada	1995	≤2.5	3–6	≥6.5	14 032	20 964	35 771	1.50	2.50
Dusankova [26], Czech Rep.	2007	0–3.5	4–6.5	7–9.5	12 512	17 765	28 902	1.42	2.31
Henriksson [35], Sweden	1998	≤3	3.5–6	≥6.5	18 966	36 818	92 861	1.94	4.90
Karampampa [38], Canada	2009	0–3	4–6.5	7–9	26 155	39 545	66 143	1.51	2.53
Karampampa [37], France	2009	0–3	4–6.5	7–9	19 041	34 792	52 320	1.83	3.75
Karampampa [40], Germany	2009	0–3	4–6.5	7–9	26 795	50 521	81 331	1.89	3.04
Karampampa [39], Spain	2009	0–3	4–6.5	7–9	29 819	63 433	85 660	2.13	2.87
Karampampa [42], UK	2009	0–3	4–6.5	7–9	18 485	40 424	92 135	2.19	4.98
Karampampa [41], Italy	2009	0–3	4–6.5	7–9	29 512	54 300	52 020	1.84	1.76
Karampampa [43], Netherlands	2011	0–3	4–6.5	7–9	37 199	61 388	120 801	1.65	3.25
Orlewska [57], Poland	2002	<3.5	4–6	>6.5	15 720	22 391	26 497	1.42	1.69
Palmer [58], Australia	2010	1–3	4–6	6.5–9	24 389	39 491	43 793	1.62	1.80
**Mean (SD)**					22 719	40 153	64 853	1.75 (0.26)	2.86 (1.09)

The studies by Kobelt and colleagues represented about one third (10/29) of all included studies in our study. These studies used similar methodology but adapted to the different country settings: Inpatient care was used by between 6.7 to 25.8% of the included patients, sickness absence was used by 4.6 to 25%, and permanent reductions in productive work due to MS concerned 32.9 to 44.5% (Table 6).

**Table 6:** Resource use in studies by Kobelt and colleagues, recalculated to 12 months.

Country studied by Kobelt et al 2006	In-patient care (% of all)	In-patient days, average by hospitalized	Sickness absence^1^ (% of all)	Sick-leave days (short term) among those with sickness absence^1^	Early retirement due to MS (% of all)
Austria	25.8	27.0	25.0	17.3	44.5
Belgium	19.0	27.5	8.8	17.0	32.9
France (2009)	17.0	15.5	11.0	21.5	?
Germany	24.5	21.0	11.0	19.2	33.9
Italy	15.6	19.3	22.4	10.3	33.3
Netherlands	7.9	18.2	9.5	17.3	42.2
Spain	17.0	12.3	5.5	6.0	34.1
Sweden	12.2	21.6	10.2	10.4	35.7
Switzerland	13.2	38.0	4.6	11.0	33.9
UK	6.7	18.8	8.4	13.9	44.3

^1^ The definitions of sickness absences varies between studies or is not stated at all

## Discussion

There were large methodological variation between the identified studies and both costs and cost drivers appeared to be influenced by methodological choices. The main methodological differences were in the inclusion of different types of costs rather than the used perspective, as most studies used a BU approach and reported prevalence-based COI estimates. Moreover, implementation and categorization based on severity level differed largely between studies, Although absolute costs differed between studies, it appears that the cost ratios between different severity levels within studies were more stable, almost as 1 to 2 to 3 for EDSS I, II, and III, respectively. Our findings also suggest that cost drivers differ by severity level, where most studies showed that drug costs dominated in lower severity levels, while the main cost drivers in more severe levels of MS were production losses and informal care.

### Methods of calculation affects the comparability between studies

Of the included studies on COI of MS, approximately 80% were published in the year 2000 or later, which indicates that studies of COI of MS can be described as a topic of recent and increasing interest. All included studies had a prevalence approach which can be used e.g., as a first step for calculations of cost effectiveness [19] of for example new drugs. However, if preventive interventions are in focus, an incidence approach would be more accurate [19, 21] to allow examination of costs over time [53]. An alternative method would be to create models based on retrieved or already published costs [68], and in two included studies such results were presented e.g., for 20-years [53] and life-time costs [33], in addition to the prevalence-based costs. Such analyses assume that the patterns of costs for different patients, age groups, and severity levels, based on a prevalence approach, can be used to estimate development in incidence-based cost over time.

According to our results from the 29 studies that were included for cost comparison, the one study using a TD approach reported the lowest mean cost per patient. It seems reasonable that the higher estimated COI for MS are, at least in parts, due to more cost components included in the calculation in BU studies. For instance, certain data may not be available in databases and by using the TD method, important cost data may be missing, e.g., costs related to complications where the disease of interest is not the main diagnosis [69]. Moreover, a potential reason for high costs when using a BU method is double counting of costs including the disease under study and its co-morbidities [69]. For instance, if some of the reported resource use assigned to MS actually were caused by a frequent co-morbidity to MS, such as depression [7, 70] the estimated COI will represent the costs for both MS and part of the costs for depression. Thus, a potentially estimated sum of costs for all diseases in a population may end up being higher than the total cost [71]. It would be preferable if future studies on COI of MS—as well as of other diagnoses—specified how resource utilization due to comorbidity was separated from that of the main diagnosis under study, in this case MS. Some comorbidities are independent from the studied disease, others a result of it. There are other examples of diseases for which it is not obvious if the COI for e.g., treatment of other diseases should be seen as related to the studied disease. One such example is diabetes where comorbidity has seen to incur high impact on total costs of illness [72].

Thus, it appears that the approaches of calculating costs of the included studies were not the main methodological differences, as all studies used the prevalence approach and almost all studies were BU. However, large variations were found regarding which costs that were included and in the handling of level of severity. Moreover, these aspects appear to be interlinked in their effect on cost drivers and overall costs.

Over time, it has become increasingly common to assess MS costs according to disease severity (i.e., EDSS). Our finding that costs increase with increasing disability level is consistent with the results of previously conducted reviews of COI of MS [7, 9–11]. Similar to the findings by Naci and colleagues [7], many studies showed that medical costs corresponded to a greater proportion at a lower severity level, while the proportion of non-medical direct costs and indirect costs increased with severity of disease. The relative relationship between costs in different severity levels has been examined previously [10, 11], whereas a review including ten studies (the most recent published in 2002) showed that the relative costs were more consistent, since the absolute costs depend on a number of contextual factors [10].

Knowledge of the different distributions of cost components between severity levels has implications for studies of MS treatments. Due to how patients are recruited to e.g., clinical trials of new treatments, a large proportion of the included patients may have a mild disease severity level and recently diagnosed MS, or have had complications to previous treatments. If patients with less severe disease are dominant, this distributional effect will result in the estimated costs for drugs being overestimated as compared to other cost components. It is thus difficult to conclude, based on such studies what the possible savings for introducing new drugs in the population are. Moreover, the health status of a patient having MS may possibly in a longer perspective deteriorate, which should be associated also with the effectiveness of given treatment, and thus result in higher indirect costs. Although other research methods are needed to estimate the economic impact of insufficient treatment effects over time, our results indicate that costs per patient may double and later triple by EDSS level.

Contextual differences may cause comparisons of COI studies of MS unfeasible, such as differences in categorization of costs and resource use. It has e.g., been argued that there might be contextual differences for patients with MS to rely on family members or on friends for informal care, which can also affect the calculated cost for informal care [51]. As the included studies categorized informal care as an unspecified direct cost [27, 34, 35, 52, 53], as a direct non-medical cost [37–51, 53], presented separately from direct and indirect costs [58] or, as an indirect cost [26, 57], Included cost categories, as well as e.g., proportions will differ due to methodological choices.

Moreover, laws and attitudes towards use of social insurances such as full-time or part-time disability pension or early retirement for patients with MS differs between countries [27], as well as how sickness absence and disability pension are described and/or measured. For instance, it is often not clear if ‘short-term sickness absence’ means e.g., <7 days or <90 days. Also, disability pension or what in some studies is called early retirement or early retirement due to MS, is seldom clearly defined. There is probably a difference between taking old-age pension early due to health problems and being granted disability pension. Previous reviews on sick leave and disability pension have pointed out the lack of clarity in how different concepts are used [73, 74]. There are also variations in organization of healthcare which may lead to differences in consumption of care but also in prices for resources of healthcare [25]. Applying estimates from studies in one population that differs from the population of interest may also cause problems for comparisons [8], as well as differences in cut-off points used for the EDSS [52]. In addition, the length of study period, e.g., one month compared to six months, may lead to inaccuracies or biases when the results are multiplied up to annual costs [61].

Furthermore, above described methodological and contextual differences probably have had an impact on what could be considered as the main cost driver. The severity of MS might differ within patient groups which add to differences of resource utilization making healthcare costs skewed [75]. Age, disability (EDSS), and presence of depression have been found to be independent predictors of costs in univariate analyses [59]. Furthermore, differences between studies as regards the proportion of patients with MS relapses of severity, as well as duration of relapses, are important for the estimated economic burden of MS [7], and differences in sampling of patients for inclusion COI studies may also be of importance for comparison between studies [50]. Questions in need of answer in connection to the (more common) BU method are several: were the patients included representative of the MS population, were the resource use reported by the patients accurate (e.g., do patients to the same extent report hospitalizations and use of over-the-counter medications during the last year or month), as well as correctly interpreted [52]. Although the same methodology was described, the response rates generally were low and differed between 16 to 52% in the nine studies by Kobelt and colleagues [25, 44–51], which could be a source of bias and affect the reliability of results [35].

What could be done to promote comparability of COI studies? One suggested solution is to give support to well-designed multinational prospective studies that could enhance the understanding of possible differences between studies [76]. An important step would be to ensure that included costs and concepts are clearly defined and described in relation to the healthcare system under study, so that readers are able to judge the applicability of results to their own settings.

### Strengths and limitations

This systematic review used a novel approach by step by step selection of more similar studies for comparison, thus indication in each step the remaining methodological differences. To our knowledge, this is the first review of the COI of MS using this approach.

The mapping of published studies that describes methods used for calculations of COI of MS made the analysis of comparing costs feasible, enabling comparisons between the COI studies of MS based on cost ratios for severity of MS according to the EDSS index. However, the mapping only entails some of the methodological differences and we acknowledge that the comparison between disease severity levels within studies provides more information than the comparison between studies. In previous reviews [10, 11], the relative relationship has been studied by using the approach of examining each disability level on the EDSS scale in relation to the average of the lowest EDSS category, which differs from our approach of comparing between mild, moderate and severe MS. This categorization of the EDSS was implemented to facilitate comparison between studies also when costs were not reported on every EDSS score in all studies. Although the cut offs for EDSS were not defined by exactly the same EDSS scores in the twelve studies included in that comparison, ratios of costs tended to increase at a rather similar rate as 1 to 1.75 to 2.86, or almost as 1 to 2 to 3, from the least to the most severe group of MS disease, and with limited deviations between the cost ratios of included studies. Although not possible to extract from the series of studies by Kobelt and colleagues [25, 44–52], these studies stated clearly that costs were greater following higher disease severity, which further emphasizes the relevance of this association between costs and severity of MS.

The full-text reading was independently performed by two authors. One limitation of the literature search was that the title and abstract examination was performed by one author, thus in this stage not having the possibility to discuss potential disagreement of relevant studies with another author. However, all studies mentioning economic burden related to MS were included for full-text reading. In order to find possibilities of comparisons, the included studies in our review were based on data from OECD-countries. This fact and the finding that 59% of the included studies were undertaken by two groups of authors with connections to research in Sweden might be interpreted as a kind of bias. However, as comparisons of outcome of ratios of costs of EDSS-levels (Table 5) show, there were high resemblances of outcome between all included studies. The restriction to OECD-countries was a consequence of the method chosen for recalculations of costs i.e., using the PPP of the OECD [23], which facilitated comparison between study results. The highest and lowest cost estimates were reported by two of the studies that were handled differently in the PPP calculations (Fig 2), which indicates the difficulties in translating costs over time and between years. After excluding these two studies, the average total cost per patient and year ranged from USD 24 666 to USD 51 678 (PPP). Furthermore, the choice of annual inflation rate will impact the comparison between studies using different years of price level, where a higher rate would reduce differences of the average total cost between compared studies.

Due to the exclusion of intangible costs in this review, we underestimate the economic impact of MS in society. According to previous research, intangible costs represented up to 50% of the overall costs of MS [14] depending on methodological choices in the original publications. Among the studies included in our study comparison, only eleven studies [25, 35, 44–52] included intangible costs. Additionally, as our findings were based on studies using mainly patient questionnaires and self-selected samples, further studies of costs resulting from MS in representative population-based samples are warranted.

## Conclusion

Although similar perspectives were applied used in the included studies, our findings support the raised concern of that results regarding the total cost per patient and year varies greatly between studies, and this was mainly due to differences in which costs that were included and how severity of disease was handled. As expected, the total costs increase with higher level of disease severity. However, we also found that the distribution of cost components varied with severity level. Although great variations between studies were found in terms of absolute costs, the relative costs expressed as cost ratios comparing different levels of severity level of MS indicated resemblances, appearing to make comparisons between studies feasible.

## Supporting Information

S1 Table Reason for exclusion in the second assessment, based on the stated inclusion criteria (n = 19). (DOCX)

S2 Table PRISMA Checklist. (DOCX)
