# Supplementary material for: Cost of Illness of Multiple Sclerosis - A Systematic Review
Source: PLoS One. 2016 Jul 13;11(7):e0159129. doi: 10.1371/journal.pone.0159129 (PMC4943600; doi:10.1371/journal.pone.0159129)
Supplement: S1 Table — (DOCX) [file pone.0159129.s001.docx]

S1. Reason for exclusion in the second assessment, based on the stated inclusion criteria (n=19)

| **First author, year** | **Reason for exclusion** |
| --- | --- |
| Amato [3], 2002 | Year of price level missing |
| Asche [1], 1997 | Number of patients missing |
| Asche [62], 2010 | Special aspect: Incidence for one year |
| Berto [2], 2011 | Direct costs only |
| Blumhardt [66], 1996 | Not human capital approach |
| Bourdette [29], 1993 | Direct costs only |
| Carton [31], 1998 | Direct costs only |
| Casado [63], 2007 | Intangible costs |
| Coleman [32], 2013 | Indirect costs only |
| Gilden [65], 2011 | Direct costs only |
| Grima [5], 2000 | Not human capital approach |
| Henriksson [4], 1998 | Number of patients missing |
| Holmes [36], 1995 | Description of calculation of indirect cost missing |
| Murphy [55], 1998 | Not human capital approach |
| Oleen-Burkey [56], 2012 | Not a societal perspective |
| Parisé [67], 2013 | Special aspect: Cost of relapse |
| Patti [30], 2011 | Direct costs only |
| Svensson [60], 2014 | Special aspect: inclusion of patients with spasticity |
| Zettl [64], 2013 | Special aspect: Cost of relapse |
